# Experimental Cold-Cured Nanostructured Epoxy-Based Hybrid Formulations: Properties and Durability Performance

**DOI:** 10.3390/polym12020476

**Published:** 2020-02-19

**Authors:** Mariaenrica Frigione, Mariateresa Lettieri, Francesca Lionetto, Leno Mascia

**Affiliations:** 1 Department of Innovation Engineering, University of Salento, 73100 Lecce, Italy; francesca.lionetto@unisalento.it; 2 CNR-SPIN, 84084 Fisciano (Salerno), Italy; mariateresa.lettieri@cnr.it; 3 Department of Materials, Loughborough University, Loughborough LE11 3TU, UK; l.mascia@lboro.ac.uk

**Keywords:** cold-cure, epoxy resins, organic-inorganic hybrids, sol-gel

## Abstract

Different hybrid epoxy formulations were produced and cold-cured, monitoring the properties development during low temperature curing and aging. All systems were based on silane functionalized bis-phenol A (DGEBA) resins (Part A), cured at ambient temperature with two amine hardeners (Part B). The different components of the formulations were selected on their potential capability to bring about enhancements in the glass transition temperature. The durability of the produced hybrids was probed in comparison to the corresponding neat epoxies by monitoring changes in glass transition temperature (*T*_g_) and flexural mechanical properties after exposure to different levels of humidity and immersion in water and at temperatures slightly higher than the local ambient temperature, in order to simulate the conditions encountered during summer seasons in very humid environments. The thermal degradation resistance of the hybrid systems was also evaluated by thermogravimetric analysis.

## 1. Introduction

Polymer-based nanocomposites have attracted a great interest over the last few decades due to their highly enhanced mechanical, electrical, thermal, barrier, and optical properties, derived from synergistic combinations of polymer matrix and inorganic nano-dimensional reinforcing and thermal resistant interpenetrating domains. From this advantageous combination new materials can be produced, exhibiting enhanced properties that can be tailored according to application requirements [1].

This class of polymeric nanocomposites is usually referred to as organic-inorganic (O-I) hybrid materials to denote a chemical and morphological structure consisting of nanoscale repeating units between the organic and inorganic constituents of nanoscale dimensions [2]. In such nanostructured systems, both the inorganic nano-dispersed component and the polymeric matrix are produced concurrently by the sol-gel method, consisting of hydrolysis and condensation reactions to produce three-dimensional interpenetrating networks. The very high surface area to volume ratio of the inorganic and organic components is considered a major factor responsible for the enhanced properties, which have successfully exploited in many applications, such as in the field of structural adhesives [3].

Many structural adhesive bonding applications make use of thermosetting resins (very often epoxy type), where curing reactions are carried out at cold, either “on field” or the products are too large to allow the use of high temperatures from typical sources of heat (ovens, lamps). Typical applications of cold-cured epoxies include: i) Structural adhesives employed either in construction (resins for injection) and in aeronautical/aerospace (“patch repair” technique) industries, and ii) matrices/adhesives for fiber-reinforced polymer (FRP) components to be used for the repairing and strengthening of infrastructures [4,5].

As well, testified by different papers that appeared in the last decade [6,7,8,9,10,11], cold-cured adhesives display intrinsic weaknesses, not only as a consequence of a hardening process carried out at cold and under uncontrolled temperature conditions but also due to adverse weathering effects on properties. Only moderate glass transition temperatures (*T*_g_) can be achieved with such cold-cured resins: Never greater than 55–60 °C even after very long curing times (in the order of several weeks), which can be further reduced through the plasticization effect of liquid water or moisture in the atmosphere under outdoor service conditions. These aspects raise great concerns about the long-term performance of such cold-cured resins, even though these resin systems are already employed in many structural applications as cold-cured epoxy adhesives and matrices for fiber reinforced composites. For such applications, therefore, cold-cured polymer-based nanocomposite materials can offer significant advantages over commercial resins [3,12].

Nanomaterials are not “unknown” in the construction industry. For instance, it has been shown that concretes can be engineered by the incorporation of nanosized building blocks (e.g., nanoparticles and nanotubes) to enhance their performance [13,14,15]. It is also possible to graft molecules onto cement particles, cement phases, and aggregates (including nanosized additives) to provide surface functionality, which can be adjusted to promote specific interfacial interactions.

In this framework, nanostructured hybrid systems, based on common epoxy resins containing interpenetrating silica nanodomains, are expected to offer superior thermal (especially in terms of glass transition temperature), mechanical, and adhesive properties and greater durability against moisture, temperatures, hash environments, and fire over the commercial products. They are also expected to provide enhanced mechanical properties in comparison to the pure polymeric matrix due to the reinforcing effect of the inorganic nanodomains. Furthermore, the latter feature is likely to promote the adhesion to the typical substrates encountered in infrastructures (concrete, masonry, steel, wood, etc.). The use of such new cold-cured resins in the construction industry, therefore, would allow improvements in performance of existing constructions (buildings, bridges, galleries, etc.) by avoiding the demolishment and reconstruction of new ones. The use of nanomaterials as modifying agents, therefore, would be make it possible to extend the longevity of the structures for longer periods.

O-I hybrids, characterized by a morphology consisting of co-continuous silica-domains in the region of 5–20 nm of organic chains chemically linked to the inorganic phase, are obtained through the sol-gel method [16,17,18,19,20]. Usually, the first step in the production of the organic-inorganic hybrids is constituted by the silane-functionalization of the epoxy resin. Then, hydrolysis reactions of the functionalized epoxy with an appropriate alkoxysilane component takes place in presence of water. The final step of the synthesis of organic-inorganic hybrids is the simultaneous cross-linking of the (organic) epoxy-based component of the mixture, upon the addition of a suitable curing agent, and the condensation of the (inorganic) siloxane domains, leading to an in situ production of silica. In the case of cold-curing epoxy-based hybrids, the latter two processes both occur at ambient temperature.

In previous studies, different epoxy-based nano-silica hybrids were produced [21,22,23]. In this paper, the physical properties and durability characteristics of the hybrids developed in previous studies are reiterated, adding also unpublished results, highlighting at the same time differences between various new epoxy-based organic-inorganic hybrids. Although based on typical bisphenol epoxy resins, the O-I hybrid systems in this work differed for the nano-silica contents, as well as for the hardeners and the molar ratios. At the same time, changes are made regarding the alkoxysilane ingredients as precursor for the silica component and the procedure used for the pre-functionalization of the resin. Particularly novel is the use of a specific deep eutectic solvent (DES) as an auxiliary component for property enhancement: To the best of our knowledge, DES systems have not been used hitherto for epoxy hybrid compositions. The research investigated also the effects of different curing agents, both suitable for cure at ambient temperature, including the addition of an accelerator, and ammonium molybdate, as a siloxane network enhancer. The ingredients for the different hybrid systems were carefully selected with the view to reduce the curing times and improve the physical properties, especially the glass transition temperature for cure performed at ambient temperature. In particular, the present study compares the characteristics of different formulations, highlighting the effect of any single component on the final cured system. 

## 2. Experimental

### 2.1. Materials and production of Hybrid and Non-Hybrid Formulations

**Scheme 1:**
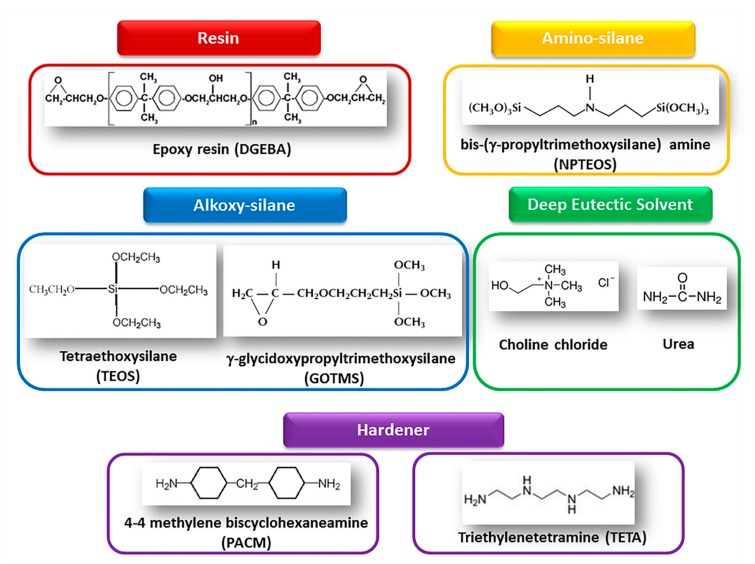
Chemical structures of all the components employed.

Different experimental two-part formulations, whose compositions are detailed in Table 1, were produced using the following materials and procedures. In Scheme 1, the chemical structure of all the components employed are summarized.

**Scheme 2:**
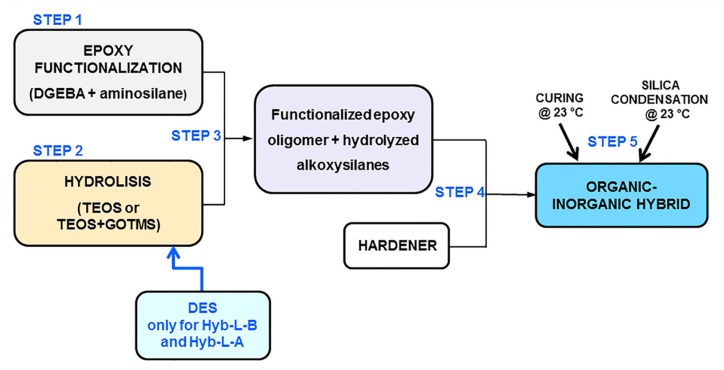
Schematic presentation of steps involved in the production of the epoxy-silica hybrids.

In Scheme 2, the main steps that were followed for the production of the hybrids of this study are illustrated. 

All systems were based on typical bis-phenol A (DGEBA) resins. For the systems Control B0, BSi and BSiMo, a resin commercially known as NPEL 128E, obtained from Nan Ya Epoxy Resins was employed, characterized by an epoxy equivalent weight of 184–190 g. The remaining systems were produced with Epikote 828 (Resolution Performance Products), possessing the same epoxy equivalent value (i.e., 184–190 g).

Step 1: Silane functionalization of epoxy resin. The procedure used has been described elsewhere [24].

For the systems BiSi and BiSiMo, an amine-trialkoxysilane (supplied by Aldrich with a purity greater than 95%) was employed as a coupling agent, as well as an alkoxysilane possessing a high functionality, again supplied by Aldrich, as experimented in a previous study [24]. For the production of the Hybrid DGEBA, Hyb-L-A and Hyb-L-B systems, the bisphenolic resin was functionalized with bis-(γ-propyltrimethoxysilane) amine, commercially known as NPTEOS and purchased by Aldrich (purity>90%). The production of Hyb-L-A and Hyb-L-B systems is the subject of a patent [25]. The resulting compounds constituted Part A for each two-part hybrid formulation.

Step 2: Hydrolysis of alkoxysilane precursors.

The second step consisted of a prehydrolysis of alkoxysilane precursors. Only in the case of BiSi and BiSiMo hybrids, Tetra-ethoxysilane (TEOS, supplied by ACROS with a purity greater than 95%) was used in sufficient amounts to produce a 7.5 wt% nominal SiO_2_ content, based on the total conversion of the alkoxysilane groups in the mixture [21]. For other hybrid systems (i.e., Hybrid DGEBA, Hyb-L-B, Hyb-L-A), a mixture of tetra-ethoxysilane (TEOS) and glycidoxypropyltrimethoxysilane (GOTMS) was used, both supplied by Aldrich (at a purity greater than 97%). A molar ratio 1:0.12 of TEOS:GOTMS was used in all the three cases. For the production of Hyb-L-A and Hyb-L-B systems, a deep eutectic solvent (DES) was added in the TEOS-GOTMS mixture. DES was obtained by mixing choline chloride (ChCl) and urea (U) in a 2:1 molar ratio; the two chemicals were supplied by Iolitec GmbH (Heilbronn, Germany) with a purity greater than 97%. The content of ChCl-U was set at 2.5 parts per hundred resins (phr), corresponding to 1.5% by weight of total mixture [23].

Step 3: Mixing of prehydrolyzed alkoxysilanes and functionalized epoxies.

This was carried out by mixing for 2 h the different solutions prepared in steps 1 and 2.

Step 4: Addition of the hardener.

Two hardeners were employed to cure the different hybrid systems, both suitable for the cure at ambient temperature. The first one was a cycloaliphatic amine, i.e., the 4,4’-methylenebis-cyclohexaneamine (PACM) supplied by Aldrich. PACM amine, although requiring somehow longer cold-curing times, is reported to produce superior elevated temperature performance. In all the formulations cold-cured by PACM, a molar ratio epoxy/amine = 1:0.75 was used. This choice was derived from previous studies carried out on cold-cured epoxy coatings, where the molar ratio 1:0.75 was identified as the optimum epoxy/amine ratio for property enhancement in relation to glass transition temperature range and the stiffness of systems cured at ambient temperature [26,27]. In the systems B0, BSi, and BSiMo, a small amount (<1%) of phenylen bis-methylamine M851 accelerator (supplied by Leuna Hartze) was also added, as a possible means to speed the curing reactions carried out at ambient temperature. In the BSiMo system, ammonium molybdate powder (with chemical formula (NH_4_)_2_Mo_2_O_7_ and obtained from ACROS) and dibutyl tin dilaurate catalyst (obtained from Aldrich with a purity greater than 95%) were mixed to PACM + M851 hardener at 6.0 and 1.0 wt%, respectively, with respect to the nominal SiO_2_ content of the hybrid formulation. The addition of ammonium molybdate was expected to produce a denser siloxane network in the organic-inorganic system and to increase the *T*_g_ of the organic phase [28,29]. Molybdate salts, acting as inhibitors, have been previously added to the hybrid polymer coatings obtained via the sol-gel process for passivation of metals, steels, alloys [30,31,32,33]. It should be noted that BSi and BSiMo were proprietary systems produced by SAFE Marine Nanotechnologies (Ferentino, Frosinone, Italy), therefore some details in the compositions were not provided by the manufacturer.

The second curing agent selected for the curing at ambient temperature was an aliphatic amine, i.e., Triethylenetetramine, TETA, supplied by Elantas Italia S.r.l. (Italy) with the commercial name IG 824-K24. TETA curing agent was used without further modification. In all the systems cured with TETA, a stoichiometric (i.e., 1:1) molar ratio epoxy:amine was used.

The unmodified or modified curing agents constituted Part B of the hybrid systems, as well as for the control formulations.

Step 5: Cold-curing of hybrid (and non-hybrid) formulations.

Irrespective to the formulation examined (listed in Table 1), Part A and Part B were manually mixed at ambient temperature for a maximum of 30 min in order to avoid premature gelation. The liquid formulations were, then, poured in Teflon molds possessing different dimensions, depending on the standard tests to perform on them. The specimens were cured at ambient temperature in a controlled environment (23 ± 2 °C and 55% ± 5% relative humidity) for prolonged periods up to one year. The long-term cold-curing process was carried out on “free” specimens, i.e., they were all removed from the mold after few days of curing. During this last step of the process, the cross-linking reactions of the epoxy component and the nano-silica condensation of the inorganic phase are taken to an advanced stage, producing an interpenetrating network where organic and inorganic phases are interconnected at nanosize level.

The nominal SiO_2_ contents of the cold-cured epoxy-siloxane hybrids are summarized in Table 1. For comparison purposes, an epoxy-based non-hybrid control formulation, representative of commercial cold-cured resins, was realized for each hybrid system.

### 2.2. Characterization of Hybrid and Non-Hybrid Formulations in Standard Conditions

The first parameter analyzed on all hybrid systems was the curing time. The effect of this parameter on different physical properties developed by organic-inorganic epoxy-silica formulations was, in fact, assessed, taking as reference the control non-hybrid systems.

As already explained, the main drawback of conventional cold-cured resins is the relatively “low” glass transition temperature, even after very long curing times. The evolution of the *T*_g_ was monitored by differential scanning calorimetry (DSC), using a Mettler Toledo DSC 822, on all the produced hybrid systems during the cold-cure process performed in a controlled environment. Curing times up to a year were analyzed. Dynamic DSC scans were carried out at a heating rate of 10 °C/min, from sub-zero temperature up to 300 °C. The specimens (with thicknesses not greater than 0.8 mm and an average mass of 10–14 mg) were placed in aluminum pans and analyzed under nitrogen atmosphere (flow rate: 60 mL/min), in order to avoid the occurrence of any undesirable oxidation reaction. The calorimetric runs were performed on three samples of each formulation and the results were averaged. The DSC analysis allowed to calculate also the residual heat of reaction after different cold-curing periods.

Flexural tests and dynamic mechanical thermal (DMTA) analysis were used to characterize the cold-cured hybrid materials. The flexural properties were measured in a three-point bending mode, using a LR5K Lloyd Instruments Machine according to the ASTM D790 standard [34] on rectangular specimens (100 × 10 × 4 mm^3^), with a span/thickness ratio of 16:1, and at a rate of 1.7 mm/min. The systems analyzed were cured in controlled laboratory conditions for four months. The effect of shorter curing times was also assessed on some of the produced systems. At least five specimens were tested for each analyzed composition. According to Equations (1) and (2), the flexural strength (*σ*_fl_) and flexural modulus (*E*_fl_) were calculated as:

(1) $$\sigma_{fl}=\frac{3\times F_{\max}\times L}{2\times b\times h^{2}}$$

(2) $$E_{fl}=\frac{F\times L^{3}}{4\times b\times h^{3}\times \delta }\;$$

indicating with *F*_max_ (N) the load at failure, L (mm) the span length, *F* (N) and *δ* (mm) the actual load and its displacement below the elasticity limit, respectively, *b* (mm) and *h* (mm) the width and the height of the tested specimen, respectively.

Dynamic mechanical thermal analysis was carried out on standard samples (40 × 10 × 1 mm^3^) employing an ARES Rheometer (Rheometric Scientific) in the rectangular torsion configuration. The DMTA tests were performed in the scanning temperature mode, using a constant heating rate of 2 °C/min, in the range 30–150 °C, at a constant amplitude (1%) and frequency (1 Hz). Samples of Control and Hybrid DGEBA systems were analyzed in a DMTA module after a seven-month cold-cure carried out in controlled conditions. DES-based hybrids, and their respective controls, were cold-cured and aged for four months in the same controlled conditions. The tests were run in triplicate on each formulation and the results averaged for measurements of the storage and loss modulus values as functions of temperature. The glass transition temperature was measured as the maximum of the loss modulus (G’’) curve.

Two scanning electron microscopies (a Zeiss EVO 40 SEM instrument and an ESEM, environmental scanning electron microscope, Mod. XL 30) were used to examine the internal morphology of fractured specimens. The specimens analyzed were fractured after a 1-min immersion in liquid nitrogen. The internal surface of some samples fractured during the flexural tests were also examined. The ESEM analyses were performed on samples without metallization, in “low vacuum” mode, with a pressure of 0.6 Torr, a beam accelerating voltage of 25 kV, and a working distance of 10 mm; secondary electron (GSE) detector was used. Energy-dispersive X-ray spectroscopy (EDS), coupled to the ESEM microscope, was applied to perform qualitative/quantitative elemental analyses on some of the produced hybrids. The spectra were collected in spots (live time 30 s) and the related results are reported as the average on five spectra. The EDS spectra were processed using the software Genesis Spectrum (version 6.2, EDAX Inc., Mahwah, NJ, USA).

### 2.3. Characterization of Hybrid and Non-Hybrid Formulations After Aging in Severe Environmental Conditions

One of the expected advantages of the hybrid cold-cured epoxy-based resins is their higher durability. In this study, this characteristic was determined by studying the effects of severe environmental conditions on both *T*_g_ values and mechanical properties.

The flexural properties were measured on the produced cold-cured (B0, BSi, BSiMo) specimens after their exposure to different levels of humidity (varying from 55% to 100%) for different times, up to three months. Before the exposure/immersion tests, the specimens were cold-cured in air for one month at ambient temperature and, then, dried to a constant mass for one additional month. The latter step was performed in a desiccator containing silica gel (corresponding to 10%–15% R.H.) for a total of cold-curing/aging period of two months. On the same aged specimens, the *T*_g_ values (from the DSC analysis) were also recorded as a function of exposure/immersion time.

Mechanical tests in flexural mode were also performed on cold-cured Hybrid DGEBA after exposure to moisture/immersion in water, as well as employing a testing temperature slightly higher (i.e., 50 °C) than the laboratory temperature, taking as a reference the control system. The choice of this test temperature was made based on a previous experiment performed during summer in Lecce (Italy) on a concrete component with the surface exposed to radiation of sun, in line with the typical applications of such cold-cured epoxy resins, such as adhesives for concrete in a Mediterranean climate. The temperature of this climatic condition for both inside and outside faces of the concrete component can surpass 50 °C with the temperature of air around 40 °C [35]. All the specimens of Hybrid DGEBA systems were cured at ambient temperature in a controlled environment (at 23 ± 2 °C and 55% ± 5% R.H.) for at least four months, in order to perform the experiments on a stable system.

For the mechanical tests carried out after different aging regimes, the specimens of Control and Hybrid DGEBA were exposed to a relative humidity of 75% ± 5% in a climatic chamber or immersed in distilled water for prolonged times (up to about six months), and subsequently tested in flexural mode. The results of flexural tests performed on the hybrid/control systems after the described aging procedures were, then, compared to those measured on unaged specimens cold-cured for the same time.

Finally, the thermal oxidation characteristics, as an indication of the fire behavior of the resins, was assessed by thermogravimetric analysis (TGA) on the hybrid/control Hyb-L-A and Hyb-L-B systems (four-month cure), employing a TGA/DSC1 (Stare System, Mettler Toledo). The samples, about 12–15 mg in weight, were placed in alumina pans and tested in air atmosphere from laboratory temperature (about 23 °C) up to 800 °C, at a heating rate of 10 °C/min. The TGA experiments were carried out in air to measure the decomposition temperatures of the produced systems to obtain an indication of the thermal oxidative resistance of the resins in a fire event.

## 3. Results and Discussion

### 3.1. Evolution of Thermal Properties during the Cold-Curing

**Figure 1:**
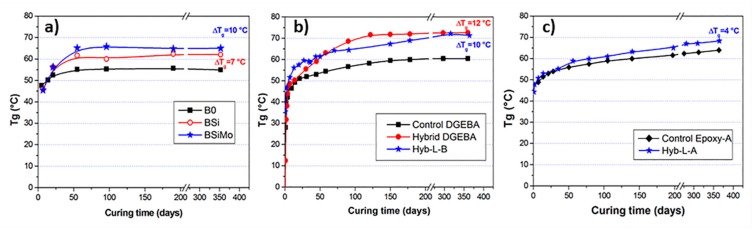
Glass transition temperature (*T*_g_) values at different cold-curing times for hybrid and control epoxy systems: (**a**) B0, BSi, BSiMo; (**b**) Control bis-phenol A (DGEBA), Hybrid DGEBA, Hyb-L-B samples; (**c**) Control Epoxy A, Hyb-L-A samples.

The progress of the glass transition temperature displayed by the different hybrids was monitored during cold-curing times up to a year. In Figure 1, the average values of *T*_g_ (by DSC analysis) for all the systems examined are reported as a function of the curing time at ambient temperature and in the environmental conditions previously described. The general behavior of the cold-cured systems can be characterized in terms of *T*_g_ increments during the first stage of curing, irrespective of the type of formulation. In the later stages, when a stable state is achieved, on the other hand, the behavior is strongly dependent on the composition. After vitrification any further (small) increase in the degree of crosslinking can only be brought about by the diffusion of unreacted species present in the network, possessing sufficient mobility to reach neighboring reactive sites. However, the contribution to advancement of curing of these latter reactions is very limited and, practically, the *T*_g_ can be considered to have reached its ultimate value, even though the system may not be fully cured at this stage, which is manifested as residual heat of reaction in DSC thermograms [22].

The most notable features of these data are that the *T*_g_ values are significantly higher (as indicated in the different graphs reported in Figure 1) than those found for the corresponding control resins, alongside a reduction in the cure time required to reach a constant value for the *T*_g_.

**Figure 2:**
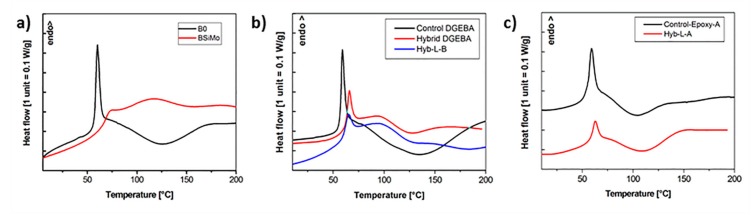
Typical differential scanning calorimetry (DSC) curves found for: (**a**) B0 and BSiMo; (**b**) Control DGEBA, Hybrid DGEBA, Hyb-L-B samples; (**c**) Control Epoxy-A, Hyb-L-A samples. All the systems were cold-cured for prolonged (greater than two months) curing times.

The lowest *T*_g_ values were registered for the systems BSi and BSiMo, reaching *T*_g_ values in the region of 65 °C after about a two-month curing time. The attainment of stable systems within a relatively short curing time (two months) confirms the advantageous role of the M851 accelerator in speeding up the crosslinking reactions. The residual heat of reaction (ΔH_res_) of the B0 system was found to decrease during the first weeks of cure and remained unchanged after curing times longer than two months. This residual heat feature was substantially not visible for the BSi and BSiMo hybrids (reported for BSiMo in Figure 2a). No exothermic phenomenon ascribable to residual cross-linking was observed in the DSC curve (Figure 2a); in addition, in the temperature range between 75 and 180 °C, a large endothermic peak, most likely due to the evaporation of alcohol formed during the hybridization of the epoxy component, can hide the residual heat of reaction. The entrapment of alcohol could have been responsible for the modest increase in *T*_g_ of BSi and BSiMo hybrids (never greater than 65 °C); nevertheless, the *T*_g_ values result are still higher than that measured for B0 control system.

It is confirmed, furthermore, the positive effect of ammonium molybdate over the unmodified resin, reflected in the *T*_g_ values for the BSiMo hybrid being higher than those exhibited by the system not containing this component, i.e., BSi. The effect on *T*_g_ of the molybdate component was explained in terms of an acceleration of the development in *T*_g_, as already observed in heat-cured hybrid systems [28]. From an applicative point of view, a crucial weakness noticed for these hybrid systems has been their short shelf life (a few months), even when they are stored at temperatures close to zero. This latter peculiarity, attributed to the effect of fast condensation reactions in presence of silanes [30,31,36], contributed to hinder a commercial exploiting of such systems. 

**Figure 3:**
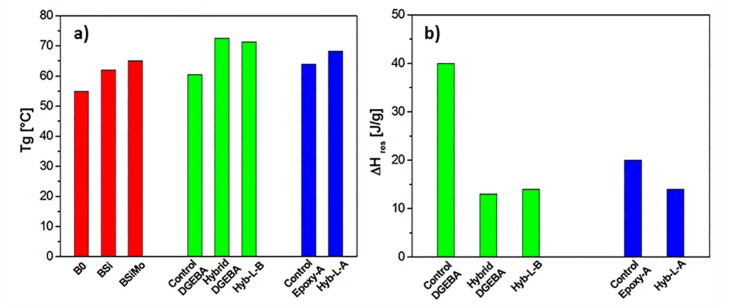
Final (**a**) glass transition temperatures (*T*_g_) and (**b**) residual heat of reaction, (ΔH_res_), measured on cold-cured systems: B0, BSi, and BSiMo; Control DGEBA, Hybrid DGEBA, Hyb-L-B; Control Epoxy-A, Hyb-L-A systems.

The Hybrid DGEBA formulation, containing neither the accelerator for crosslinking reactions nor ammonium molybdate, developed a glass transition temperature even greater than 70 °C but after a slightly longer cold-curing time, i.e., about four months. The hardener employed to cold-cure this particular hybrid was the same employed in our previously studied systems (PACM). It is worth noting that *T*_g_ values at around 70 °C are higher over the commercially available epoxy-based resins reported in the literature [37,38,39]. As observable in Figure 2b, an exothermic peak, testifying that the cross-linking reactions were not completed during the cold-cure, is clearly visible also in the thermogram of Hybrid DGEBA, even if it is appreciably smaller if compared to the control resin (see Figure 3b). On the other hand, no evaporation of alcohol can be noticed from the same curves.

*T*_g_ values greater than 70 °C were achieved also for the Hyb-L-B system, again cured with the cyclohexaneamine PACM and containing a low amount nano-silica (7 wt%), after a prolonged cold-cure (about 9–10 months). By comparing this system to Hybrid DGEBA, as illustrated in Figure 3a, it can be concluded that the addition of choline chloride-urea brings about the same increase in *T*_g_ relative to the control resin at only half the level of hybridization of the resin, even though the development of *T*_g_ seems to be slower. It should be noted that the beneficial effect of DES is achieved with only small amounts (1.5 wt%). Moreover, when compared to BSi, BSiMo hybrids, the Hyb-L-B system is capable of developing a substantial greater *T*_g_ value, even though the SiO_2_ is the same, as shown in Figure 3a. It is worth noting that the residual heat for cross-linking reactions, displayed in Figure 2b and in Figure 3b, is very similar to that measured on the system Hybrid DGEBA. Again, no endothermic peak, ascribable to evaporation of alcohol, can be observed in the DSC trace (Figure 2b).

The hybrid system containing DES and TETA aliphatic amine (i.e., Hyb-L-A) can be seen to display a *T*_g_ value approaching 70 °C after a very long curing time (about one year). It cannot be excluded, however, that the systems cured with TETA hardener, irrespective of whether it is used in the hybrid or its control neat epoxy, did not reach the stable *T*_g_ state (plateau) even after one year-curing, suggesting that it could continue to rise further at longer curing/aging times. The high *T*_g_ obtained in the Hyb-L-B system, however, can be partly attributed to rigidity of the aliphatic ring in the PACM molecule [23]. Once again, evaporation was not observed in DSC curves of Hyb-L-A system (Figure 2c); furthermore, it was possible to observe and measure a residual heat of reaction (Figure 3b), that was comparable with that measured for Hybrid DGEBA and Hyb-L-B systems.

In Figure 3, the final *T*_g_ (Figure 3a) and residual heat of reaction, ΔH_res_ (Figure 3b) measured on the cold-cured, control and hybrid, systems analyzed, are summarized.

From the previous discussion and the observation of the data reported in Figure 3a, it can be concluded that the produced cold-cured hybrid epoxy-silica resins are able to provide a noticeable increase in the glass transition temperature with respect to commercial cold-cured epoxies. This observed advantage seems to arise even in the presence of a low amount of silica (i.e., 7%) in the hybridized epoxy, but in the presence of a DES component. The chemical formulation of each hybrid, in terms of both the selected components and their amounts, has a relevant effect on the final *T*_g_ value, as well as on the time required to attain a stable system. The use of a catalyst makes it possible to achieve a stable system in shorter times at the expense of the final *T*_g_ of the hybrid.

### 3.2. Mechanical Characteristics and Morphology in Standard Conditions

In Table 2, the results of mechanical tests are summarized, performed in flexural mode. These show that in all cases the hybrids display general enhancements in both flexural strength and modulus in comparison to the respective control resin. The hybrid systems cured for two months in the presence of accelerator M851, i.e., BSi and BSiMo, however, displayed the lowest mechanical properties, even lower than those measured on the control formulations. This could be attributed to the presence of residual alcohol produced from the hydrolysis and condensation sol-gel reactions, bearing in mind the thickness of the specimens used in the tests (4 mm) and the DSC curves presented in Figure 2a. The *T*_g_ data in Figure 1, on the other hand, show that a stable high-*T*_g_ state is reached for the two-month cure period. In the latter case, the residual alcohol can escape through small openings in the nonhermitically sealed DSC cups used. 

**Figure 4:**
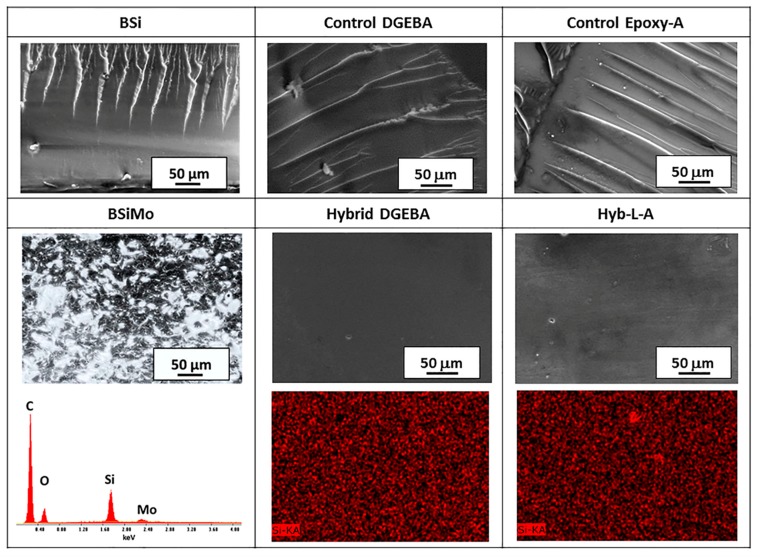
SEM micrographs of the different hybrid systems produced, compared with control systems. For some hybrid systems, EDS spectra or maps are also reported.

Nevertheless, the SEM examinations performed on the BSi system (Figure 4) revealed the presence of a homogenous structure through the entire thickness, with no occurrence of segregation of components or formation of voids. The systems produced in absence of the molybdate salt were completely transparent to the naked eye, while those containing the molybdate (BSiMo) were found somewhat opalescent, which is attributed to the presence of undissolved ammonium molybdate particles.

Appreciable improvements in mechanical properties, i.e., both in flexural strength (more than 60%) and stiffness (more than 140%) relative to the control resin, were found for Hybrid DGEBA cured for four months, due to the efficient reinforcement of the inorganic phase (siloxane domains). It should be noted that at the curing time used the *T*_g_ was in the plateau stage of the cure (see Figure 1b).

In order to assess the effect due to shorter cold-curing times, the mechanical properties of these hybrid/control systems were evaluated also after a two-month cold-curing period. The results of flexural tests, reported in Table 2, confirm that longer curing times, at least four months, are necessary for a full development of mechanical properties, particularly in the case of the Hybrid DGEBA system. Anyway, the superior performance of this hybrid epoxy-silica system, especially in terms of flexural strength, is evident even for curing times below the plateau stage is reached.

In Figure 4, SEM micrographs for Control DGEBA and Hybrid DGEBA specimens fractured in nitrogen are shown. These indicate that the control sample displays the typical topology of a brittle epoxy resin, characterized by distinct fracture lines, and the hybrid system exhibits features that are characteristic to organic-inorganic hybrids, consisting of a flat pattern with diffuse silica domains finely dispersed within the organic matrix [40,41].

The hybrid systems cured for four months and containing the DES component exhibit a general increase in strength relative to the control resin, even twofold when a cycloaliphatic curing agent was employed (i.e., Hyb-L-B system). Only small increases in flexural modulus were observed in such hybrids relative to control resins and, again, the greatest increase was measured for the Hyb-L-B system. The flexural modulus value for the Hyb-L-B hybridized resin, on the other hand, was substantially higher than that obtained for the Hyb-L-A formulation, possibly due to a combination of factors, such as the rigid ring structure of the cycloaliphatic hardener and the lightly higher silica content in the Hyb-L-B system.

The SEM observations performed on control and hybrid systems fractured during the flexural tests are shown in Figure 4. Compared to the neat epoxies (Control DGEBA and Control Epoxy A), the hybrid systems show a significantly different pattern. This is to say that the control systems displayed the typical feature of brittle epoxy materials (i.e., long fracture lines) while no fracture lines were observed in either hybrid systems. Furthermore, in the latter case, no macroscopic phase separation between organic and inorganic phases are detectable, confirming that these are interconnected at nanoscale dimension level.

The EDS analyses performed on BSi and BSiMo revealed the presence of C, O, and Si as the main elements, while traces of Mo were found in BSiMo. In both samples, a homogeneous distribution of the constitutive elements was observed. The spectra, acquired in different points, resulted as repeatable and the content in each element (including Si), evaluated as weight percent, was uniform along the specimen.

As observed from the EDS map of the Silicon distribution in Hybrid DGEBA and Hyb-L-A samples in Figure 4, the Si signal is homogenously displayed in each part of the analyzed sample. The EDS analysis thus confirms that a homogenous distribution of silica is achieved also in these hybrids.

A comparison of flexural properties measured on the different hybrid systems produced suggests that the amount of silica (reported for each hybrid system in Table 2) has a significant effect on mechanical properties. A quantitative comparison, however, is not feasible since the procedures to obtain the different hybrids are somewhat different.

**Figure 5:**
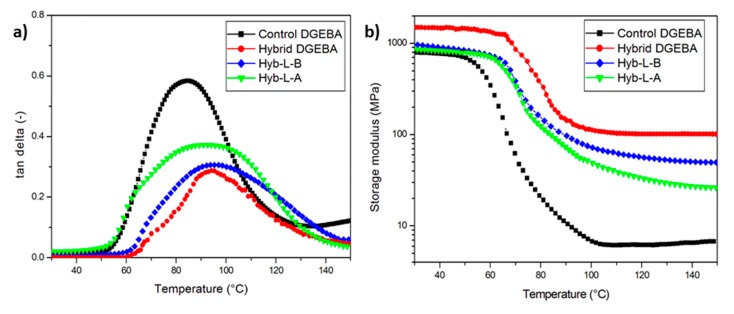
Results ((**a**) tanδ and (**b**) storage modulus) of the hybrids cold-cured in presence of PACM or TETA hardeners and of the Control DGEBA.

The results of DMTA tests performed on representative hybrid systems of the present work are presented in Figure 5.

The storage modulus values at around typical ambient temperatures for potential outdoor applications of the Hybrid DGEBA system are expectedly higher than those recorded for the unmodified epoxy resin (Control DGEBA), as evident in Figure 5b. The increase in G’ modulus upon hybridization of epoxy is even more pronounced in the rubbery-plateau region (one order of magnitude), which is accompanied by a substantial depression of the viscous losses relative to Control DGEBA. Adding these characteristics to an approximately 10 °C increase in *T*_g_ (calculated as the peak of tanδ curve, Figure 5a, the performance enhancement exhibited by these Hybrid systems can be extended to applications with higher service temperatures, such as hot water tanks, coatings or matrices for fiber composites. The modulus at 100 °C for Hybrid DGEBA is equivalent to that of a typical medium density polyethylene with an expected incremented resistance to viscoelastic creep. It is well known, in fact, that MDPE has a Young modulus in the range 0.5–1.0 GPa [42]. The presence of co-continuous elastic siloxane domains produces a very efficient reinforcement, as well decreasing the viscoelastic nature of the system.

Very similar results can be observed also for the cold-cured hybrid systems containing the DES ChCl-U, i.e., Hyb-L-B and Hyb-L-A, shown in Figure 5a,b. The siloxane hybridization of the epoxy resin containing ChCl-U produced a synergistic effect for the enhancement of the storage modulus over the whole temperature range examined at half SiO_2_ content with respect to the Hybrid DGEBA system. The increase in modulus G’ is again much more pronounced at high temperatures, i.e., the rubbery plateau region. The lower G’ values of Hyb-L-A can be ascribed to the less rigid structure of this system brought about by the aliphatic hardener compared to the cicloaliphatic hardener used for Hyb-L-B system. A rise in the *T*_g_ values, estimated as tanδ peak, is observed for Hyb-L-B and Hyb-L-A systems over the respective control resins (9 and 7 °C, respectively), alongside the depression of viscous loss at around the glass transition region. The behavior of tanδ curves indicates a hindering of the molecular motion of the organic chains brought about by the presence of silica nanodomains.

### 3.3. Properties and Performance of Hybrid Formulations in Severe Environmental Conditions

In order to assess the durability of the proposed systems when exposed to outdoor conditions, calorimetric and mechanical tests were performed on the produced hybrid systems after their exposure to environmental agents to simulate realistic weathering conditions.

**Figure 6:**
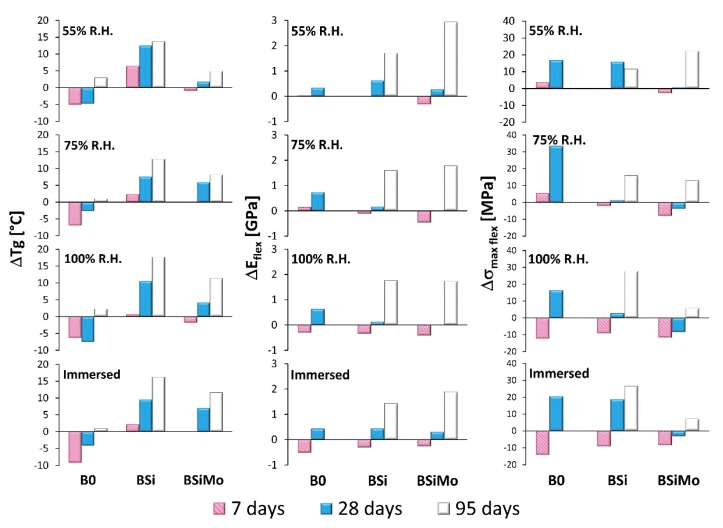
Variations in glass transition temperatures and in flexural mechanical properties (modulus and yield strength) for systems B0, BSi, and BSiMo subjected to different aging procedures for up to approximately three months.

The tests have revealed a peculiar behavior of the hybrid systems during aging in moisture/water environment. The expected deterioration in mechanical (flexural) properties, as well as in *T*_g_, due to water plasticization, typically observed for commercial epoxy resins when aged in water or exposed to high levels of moisture [8,10,43], was not found in BSi and BSiMo cold-cured formulations, as illustrated in Figure 6. Instead, an increase of the glass transition temperatures, Young modulus, and the ultimate strength was recorded upon exposure to such environments. This unexpected behavior can be explained in terms of an increase in the network density of the siloxane nanodomains formed within the epoxy component of hybrids, resulting from a continuation of sol-gel reactions during the aging under high humidity conditions. An appreciable increase in the *T*_g_ of the hybrid material is also observed with increasing in the level of humidity in the atmosphere.

After an initial decrease in *T*_g_ at shorter aging times upon immersion in water and/or exposure to 75% R.H., the Hybrid DGEBA system experienced a complete recovery of the attained *T*_g_ value after about six months aging, as shown in Table 3. Again, this can be ascribed to a continuation of sol-gel reactions activated by the absorbed water, causing further densification of the siloxane network. The superior mechanical performance of this hybrid system is confirmed from the results of the aging tests at 75% R.H. and immersion in water (see Table 3).

The results show that the plasticization effects due to the absorbed water was appreciably reduced by the silica nanodomains formed through sol-gel reactions, which hinder the long-range molecular relaxations of the organic phase. This feature represents another important advantage of epoxy-silica hybrids over conventional epoxy systems.

In Table 3, it shown also that the cold-cured Hybrid DGEBA exhibit satisfactory mechanical properties even at 50 °C, which can be attributed to the acquired higher *T*_g_ (about 73 °C). It is worth noting that the strength and the stiffness measured on this hybrid at 50 °C remained greater than the same properties displayed by the control resin (see data reported in Table 2 for Control DGEBA). This feature represents another outstanding advantage of epoxy-silica hybrids over commercial cold-cured epoxies.

**Figure 7:**
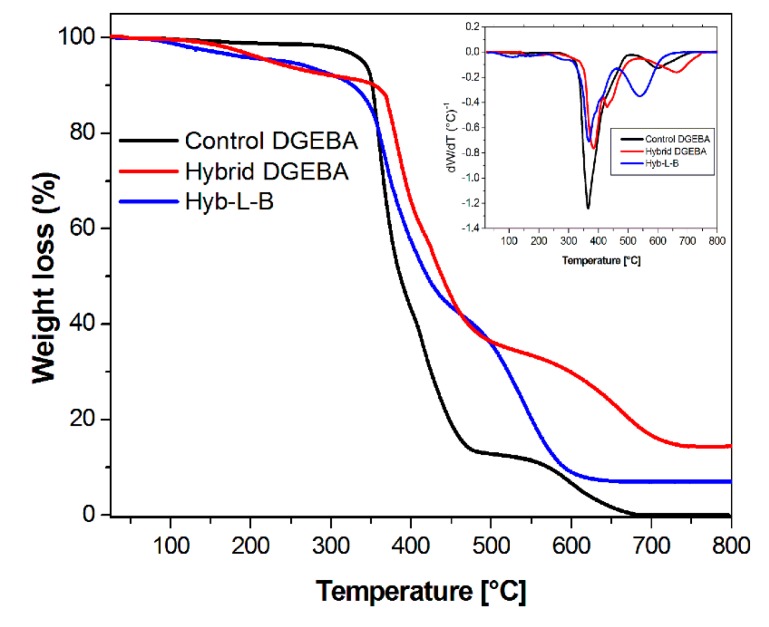
TGA curves of the hybrids cold-cured in presence of PACM amine and of the relative control. In the inset the derivative of the TGA curves obtained for the same systems is shown.

The behavior of the hybrid systems at very high temperatures is illustrated in Figure 7 with the results of thermo-gravimetric analysis performed on some of the produced hybrids.

Compared to the neat epoxy resin, the hybrid systems in Figure 7 exhibit a significant weight loss between 100 and 300 °C, due to evaporation of volatiles (water and mostly alkanols) arising from the condensation reactions within the siloxane domains. In the case of Hyb-L-B system, the observed weight loss is also due to the thermal decomposition of ammonium cations associated with DES [23].

A high thermal resistance, also relative to the control resin, can be observed for both hybrid systems (i.e., Hybrid DGEBA and Hyb-L-B). The peak temperatures of the thermal degradation process measured for the three systems are respectively 366 °C for Control DGEBA, 383 °C for Hybrid DGEBA, and 368 °C for Hyb-L-B. The upward shift of peak degradation temperature is, therefore, very significant for the system with the highest silica content (15 wt% for Hybrid DGEBA).

The observed enhancement in the thermal stability can be again attributed to the presence of the inorganic siloxane component, which form siliceous interpenetrating barrier layers to both the infusion of oxygen and the outer diffusion of volatile pyrolysis products. In concordance with the aging results, the highest the level of hybridization, the better the thermal oxidation resistance.

## 4. Conclusions

Different organic-inorganic hybrid cold-cured epoxy-silica formulations were produced and cold-cured, aiming to provide the basis for the development of new systems as efficient cold-cured adhesives for structural applications. The study has evaluated the more relevant physical properties, as well as the durability in different exposure regimes. It was found that these novel O-I epoxy-based hybrids display significant advantages over the conventional epoxy resins used as structural adhesives or as matrices for fiber reinforced composites in terms of higher glass transition temperatures, better mechanical properties and enhanced durability in aqueous environments. The investigation has examined also the effects of different amine curing agents, as well as the use of a typical accelerator (M851), ammonium molybdate, and a deep eutectic solvent. These novel systems could provide a viable solution to the well-known durability issues of the conventional cold-cured epoxy systems employed for concrete repairing and structure strengthening applications. Ongoing studies are examining the possibility of using such cold-cured epoxy-silica hybrids in conjunction with precured fiber reinforced composites or other carbon fiber-based products suitable for retrofitting applications in structural engineering.

## 5. Patent

Some of the systems presented in the paper were developed in the framework of the patent: F. Lionetto, M. Frigione “Organic-inorganic hybrids polymerized in situ at room temperature”, EP2977407 A1.

## Figures and Tables

**Table 1:** Compositions of the produced systems.

System	Resin (Part A)	Curing Agent (Part B)	Amine/Epoxy Ratio	SiO_2_ Content (%)
B0	DGEBA	PACM + M851	0.75/1	-
BSi	DGEBA	PACM + M851	0.75/1	7.5%
BSiMo	DGEBA	PACM + M851 + (NH_4_)_2_Mo_2_O_7_	0.75/1	7.5%
Control DGEBA	DGEBA	PACM	0.75/1	-
Hybrid DGEBA	DGEBA	PACM	0.75/1	15.0%
Hyb-L-B	DGEBA	PACM	0.75/1	7.0%
Control Epoxy A	DGEBA	TETA	1:1	-
Hyb-L-A	DGEBA	TETA	1:1	5.1%

**Table 2:** Mechanical properties measured in flexural mode at ambient temperature on cold-cured hybrid/non-hybrid formulations.

System	Flexural Strength (MPa)	Flexural Modulus (GPa)	SiO_2_ Content (%)
B0	46.8 ± 2.9	1.2 ± 0.1	-
BSi	16.8 ± 0.7	0.5 ± 0.1	7.5
BSiMo	20.9 ± 5.4	0.8 ± 0.1	7.5
Control DGEBA (two-month cure)	26.2 ± 3.8	2.9 ± 0.1	-
Control DGEBA (four-month cure)	30.9 ± 4.5	2.2 ± 0.1	-
Hybrid DGEBA (two-month cure)	47.0 ± 5.1	3.0 ± 0.2	15.0
Hybrid DGEBA (four-month cure)	75.2 ± 4.7	3.6 ± 0.1	15.0
Hyb-L-B	61.8 ± 2.4	2.6 ± 0.1	7.0
Control Epoxy A	42.5 ± 2.0	2.3 ± 0.1	-
Hyb-L-A	64.4 ± 4.1	2.1 ± 0.9	5.1

**Table 3:** Glass transition temperature (*T*_g_) and mechanical properties (measured in flexural mode) on cold-cured Hybrid DGEBA system after different environmental exposures.

Environmental Exposure	Testing Temperature (°C)	Glass Transition (°C)	Flexural Strength (MPa)	Flexural Modulus (GPa)
75% R.H.	23	67.7 ± 1.5	66.3 ± 8.1	3.1 ± 0.1
Immersion in water	23	70.5 ± 1.8	58.5 ± 12.5	3.0 ± 0.3
None	50	-	45.9 ± 5.5	3.1 ± 0.1
